# At the Crossroad with Morbidity and Mortality Conferences: Lessons Learned through a Narrative Systematic Review

**DOI:** 10.1155/2016/7679196

**Published:** 2016-04-17

**Authors:** Xin Xiong, Teela Johnson, Dev Jayaraman, Emily G. McDonald, Myriam Martel, Alan N. Barkun

**Affiliations:** ^1^Department of Gastroenterology, University of Toronto, Toronto, ON, Canada M5G 2C4; ^2^Department of Family and Community Medicine, University of Toronto, Toronto, ON, Canada M5G 1V7; ^3^Department of Internal Medicine and Department of Critical Care, McGill University Health Center, Montreal, QC, Canada H3G 1A4; ^4^Jewish General Hospital, Montreal, QC, Canada H3T 1E2; ^5^Department of Internal Medicine, McGill University Health Center, Montreal, QC, Canada H3G 1A4; ^6^Division of Gastroenterology, McGill University Health Center, McGill University, Montreal, QC, Canada H3G 1A4; ^7^Epidemiology and Biostatistics and Occupational Health, McGill University Health Center, McGill University, Montreal, QC, Canada H3G 1A4

## Abstract

*Objective*. To determine the process and structure of Morbidity and Mortality Conference (MMC) and to provide guidelines for conducting MMC.* Methods*. Using a narrative systematic review methodology, literature search was performed from January 1, 1950, to October 2, 2012. Original articles in adult population were included. MMC process and structure, as well as baseline study demographics, main results, and conclusions, were collected.* Results*. 38 articles were included. 10/38 (26%) pertained to medical subspecialties and 25/38 (66%) to surgical subspecialties. 15/38 (40%) were prospective, 14/38 (37%) retrospective, 7/38 (18%) interventional, and 2/38 (5%) cross-sectional. The goals were quality improvement and education. Of the 10 medical articles, MMC were conducted monthly 60% of the time. Cases discussed included complications (60%), deaths (30%), educational values (30%), and system issues (40%). Recommendations for improvements were made frequently (90%). Of the 25 articles in surgery, MMCs were weekly (60% of the time). Cases covered mainly complications (72%) and death (52%), with fewer cases dedicated to education (12%). System issues and recommendations were less commonly reported.* Conclusion*. Fundamental differences existed in medical versus surgical departments in conducting MMC, although the goals remained similar. We provide a schematic guideline for MMC through a summary of existing literature.

## 1. Introduction 

Morbidity and Mortality Conferences (MMCs) are held ubiquitously throughout medical services worldwide [1–6]. Historically, they became an integral component of surgical departments in the early 1900s, following conferences on hospital standardization [7, 8] and introduction of the “End Result System” by Ernst Codman who was first to systematically record and review patient demographics and related adverse events [3, 9]. Since the publication of* To Err is Human* [6], MMCs continue to be a widespread practice in medical training programs and are designed to “identify medical errors in order to learn from them to improve medical practice” [3].

The Accreditation Council for Graduate Medical Education (ACGME) has incorporated mandatory MMC in each training program since 1983 [10, 11]. Furthermore, the majority of hospitals require MMC in order to maintain accreditation. Over time the focus has now shifted towards identifying and correcting system-related issues through the evolving field of quality improvement, as opposed to assigning blame and responsibility to the individual [12–14].

Despite efforts to unify MMC format, their contents remain heterogeneous [1, 8], with no clear guidelines for execution. For example, there is a dichotomy of practice between medical and surgical departments [1, 5], with differing recommendations from the ACGME [10, 11]. Surgical ACGME requires weekly MMC to be performed, whereas a frequency has not been specified in the ACGME for most medical subspecialties. In addition, the case selection process for both is largely unspecified. In some studies, cases are selected from a list of voluntarily reported morbidities [15, 16], whereas, in others, they are selected from predefined complication registries [17].

The goal of this paper is twofold. First goal is to determine, through a narrative systematic review of the literature, the process and content of MMC in medical and surgical departments. Second goal is to provide a schematic guideline to improve the organization of these conferences based on the available literature.

## 2. Methods

### 2.1. Search Strategy

We performed a computerized medical literature search from January 1, 1950, to October 2, 2012, using OVID MEDLINE, EMBASE, CENTRAL, Scopus, and ISI Web of knowledge 5.6. We selected articles using a search strategy with a combination of MeSH headings and text keywords related to (1) mortality or morbidity and (2) medical education, teaching rounds, conferences, or presentation. We carried out recursive search and cross-referencing using a “similar articles” function. We also identified articles through hand searches after the initial search. We included all original studies on adult population focused on the discussion of the MMC, in French or English. Studies with original data regarding multiple aspects of MMC were assessed. We excluded articles with only abstract publication or conference presentations because these do not provide sufficient information for the purpose of this review. We reviewed national surveys, but we did not collect their data for analysis in this systematic review. Duplicates were excluded. Two investigators (Xin Xiong and Teela Johnson/Alan N. Barkun of the authors) assessed all articles according to the selection criteria independently; disagreements were discussed until a consensus was reached.

### 2.2. Choice of Outcomes and Variables of Interest

**Figure 1:**
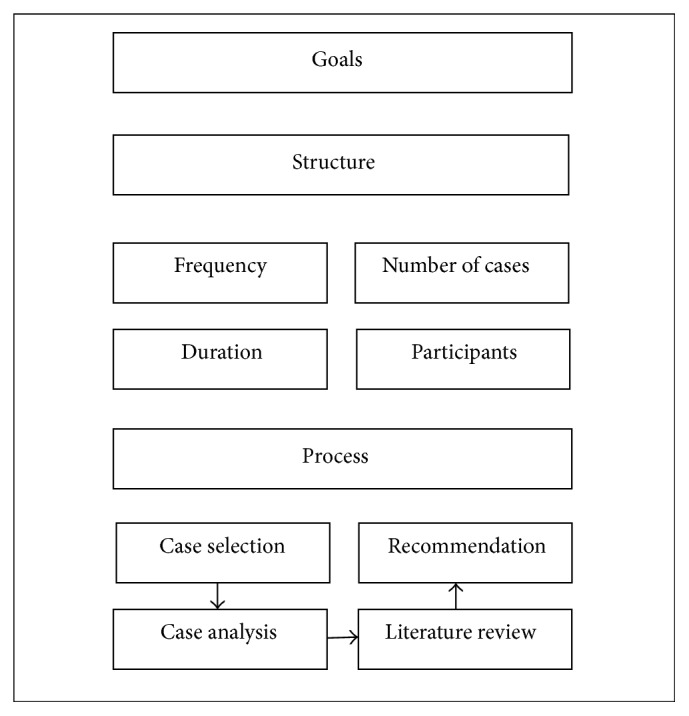
Proposed characteristics of MMC, modified with permission from Aboumatar et al. [2].

In the current literature, there exists a variety of organized terminologies to describe different aspects of MMC. In our study, we adjudicated each article's main focus into one of the following categories: goals, structure, or process [2]. For the purposes of this review, the definition for each of these categories was adapted from the following concepts described by Aboumatar et al. [2] (Figure 1).* Goal* is the objective achieved by conducting MMC.* Structure* characterizes how MMC is carried out; this includes MMC frequency, duration, number of cases presented, and participants (moderator, presenters, and audience).* Process* indicates the case selection, analysis, literature review, and proposal for improvement. Whether recommendations were implemented as a result of MMC discussion was also noted. In addition, we also collected information with respect to each study's setting, discipline, study methodology, stated objectives, and outcomes as well as how these were measured. Given that the ACGME has specified different requirements for medical and surgical specialties regarding MMC [10, 11], we collected and analyzed these data separately.

### 2.3. Sources of Possible Heterogeneity

Comparative qualitative analyses were performed across studies to assess the clinical homogeneity of study populations (cases, patients, or health care professionals), interventions, and outcomes. Statistical heterogeneity was not evaluated as most outcomes were qualitative in nature.

## 3. Results

### 3.1. Included Studies

**Figure 2:**
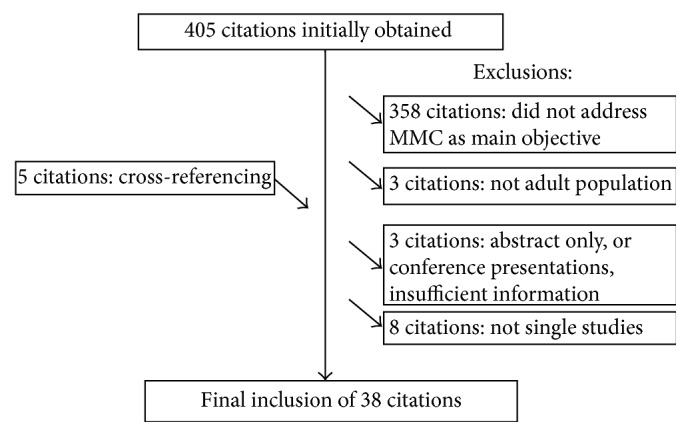
QUOROM diagram.

From a total of 405 citations identified, 358 were excluded because they did not pertain to discussion of aspects of MMC, 8 were excluded given they were either national surveys or review articles, 3 were excluded because they did not address adult populations, and 3 were excluded due to insufficient information. Cross-referencing yielded 5 additional articles. Therefore, 38 studies were included (see Figure 2).

### 3.2. Synthesis of Literature

Tables 1(a), 1(b), and 1(c) provide a summary of the 38 studies included in this narrative systematic review. Ten articles pertain to departments or divisions of medicine (including internal medicine and its subspecialties, primary care, and critical care), 25 to surgery (which includes surgery and its subspecialties, obstetrics, and anesthesia), and 3 to both medicine and surgery. These tables highlight the heterogeneity amongst studies existing in the literature. The majority of studies were performed in academic centers (34/38 or 89%): 15/38 (40%) were prospective studies and 14/38 (37%) retrospective; 2/38 (5%) used a cross-sectional design, while 7/38 (18%) were interventional. Of note, articles addressing surgical departments tended to be more quantitative than those studying medical departments. In addition, there were no uniform definitions of the various aspects of MMC (goals, structure, and process) and there was no homogenous method for measurement of errors across studies.

Overall, the focus (goal, structure, and process) that these 38 articles have covered (numbers not mutually exclusive) is as follows: 30/38 articles (79%) discussed the goal of the MMC, 30/38 (79%) the structure, and 26/38 (68%) the process. 10/38 articles (26%) discussed goal and structure, 2/38 (5%) goal and process, and 6/38 (16%) structure and process. 14/38 articles (37%) encompassed all 3 categories.

### 3.3. Medicine

**Figure 3:**
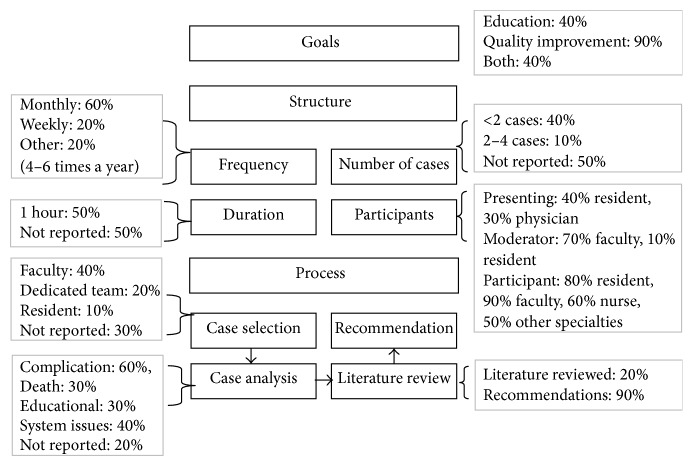
Characteristics of MMC in medicine (*n* = 10, percentages not mutually exclusive).

Figure 3 demonstrates the details of characteristics of MMC in medicine. In summary, from the review of 10 articles, the goal appeared to be quality improvement in 90% and education in 40% (percentages are not mutually exclusive). The frequency was most often monthly (60%). The duration most often spanned 1 hour (50%). Participants included faculty, residents, nurses, other health care professionals, and staff of different specialties. Usually, cases were presented by residents (40%) and less often by faculty (30%). In 70% of cases, the moderator was a faculty member. The cases were all selected before MMC, most often by faculty (40%). Cases frequently addressed complications (60%). Only 20% of articles reported a requirement of a literature review, but 90% reported implementation of recommendation. A more detailed tabular description of the rounds' content is shown in Figure 3, adopting the proposed MMC study characteristics identified in Figure 1.

### 3.4. Surgery

**Figure 4:**
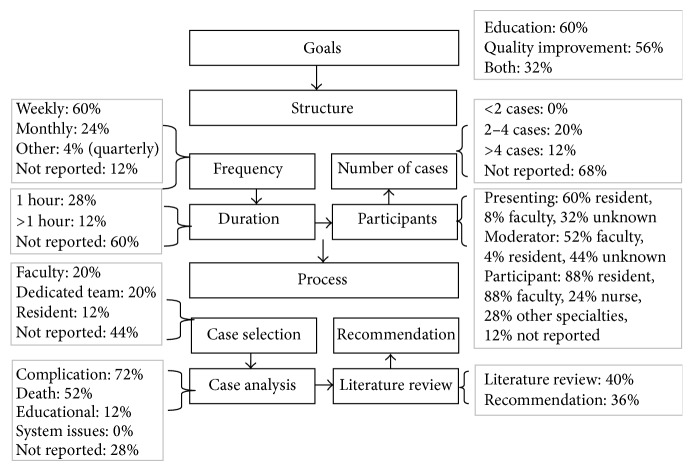
Characteristics of MMC in surgery (*n* = 25, percentages not mutually exclusive).

Figure 4 presented the details of characteristics of MMC in surgery. In summary, after reviewing 25 articles, the goals seemed to be predominantly targeting education (60%) or quality improvement (56%). The frequency was most often weekly (60%). The duration of the MMC was most often not reported (60%), but, when documented, most MMC lasted 1 hour (28%). Participants included faculty, residents, nurses, other health care professionals, and staff of different specialties. Usually, cases were presented by residents (60%). In 52% of cases, the moderator was a faculty. The cases were all selected before MMC, most often by faculty (20%) or dedicated team members (20%). Cases were selected if they addressed complications (70%), including death in 52%. Only 40% of articles reported the requirement of a literature review to support the MMC (see Figure 4).

As can be noted from Figures 3 and 4, there are differences between MMC performed in medical and surgical departments. In medical departments, MMCs are more often monthly whereas in surgery they are weekly. Medical MMCs present fewer cases, with a greater focus on discussion and analysis of systems issues, with the goal of providing recommendations for improvements.

## 4. Discussion

Upon review of the available literature on MMC, it is apparent that there is considerable heterogeneity in the content and goals of MMC across both medical and surgical services. This heterogeneity has been shown to limit the effectiveness of MMC [3, 15, 32, 42, 51]. Through the current review, we observe an important lack of standardization and precision of the definitions and terms used to describe different aspects of MMC (including goal, process, and structure). In many studies, what we believe to be important characteristics of MMC have not been recorded consistently, giving rise to only a few quality articles, leading us to believe that the reported outcomes are less generalizable. Because of this lack of rigorous reporting and poorly generalizable data, a synthesis of the literature is challenging. However, we are still able to infer several helpful conclusions regarding the process and content of MMC.

To begin with, we note that medical and surgical departments have different approaches to the process of MMC, which has been confirmed by previous reviews [1, 5, 51] and national surveys [3, 51, 52]. In medicine and its subspecialties, the goal of these conferences appears more focused towards quality improvement, whereas, in surgery, education and quality improvement are more balanced. Surgical departments comply with the ACGME MMC frequency requirement [11], with weekly meetings. In comparison, possibly because no such frequency guideline exists for [10] medical departments, MMCs are often done on a monthly basis. Moreover, surgical departments present more cases per MMC. Combining this with increased frequency, more cases are presented in surgery compared to medicine [1, 4, 5]. In contrast, discussion of fewer cases in medicine departments may allow for increased opportunities for discussion of system issues, recommendations, and follow-up of identified problems [1, 2].

There exists no direct evidence for a need for differing practices between surgical and medical departments. For example, no empiric data favors presenting more versus less cases; certain studies [17, 41, 42, 44] propose presenting all perioperative complications and mortalities, while other studies [2, 15, 45, 53] rather suggest to adopt an in-depth analysis of a few selected adverse outcomes. However, even when a majority of adverse outcomes are presented, significant evidence still suggests that MMC underreport complications as compared to other quality assurance databases, such as the National Surgical Quality Improvement Program (NSQIP) [28, 41, 42, 48]. Several reasons have been proposed [1, 2, 17, 32, 54] and include a dearth of rigorous definitions of postoperative adverse events, a lack of available resources to facilitate comprehensive data collection, and insufficient time to present all complications.

Although MMCs do not include assessment of all adverse outcomes and errors, their benefit in improving patient care has nevertheless been demonstrated quantitatively in some controlled studies: Antonacci et al. [34, 55] have demonstrated a 40% decrease in gross mortality over 4 years with rigorous reporting of cases with predefined selection criteria. Similarly, Kirschenbaum et al. [18] have reported a decrease in morbidity and mortality after instituting MMC in the ICU setting. More specifically, significant decreases have been noted in the number of cardiac arrests (3.1/1000 to 0.6/1000, *p* = 0.002) and all cause deaths (34/1000 to 24/1000, *p* = 0.024). These provide quantitative evidence of the quality improvement role fulfilled by MMC [18, 29, 32, 34]. Furthermore, the shift towards providing a safer learning environment with less individual blame [1, 6, 14, 16, 50] has encouraged increased staff and resident participation in the MMC process and has led to a more prominent role in medical education [10, 22, 31, 35, 36, 39]. These conclusions, however, are limited by the nonrandomized and qualitative study methodology seen almost uniformly across the studies we examined.

Traditionally, MMCs have consisted of case presentation by a senior resident, followed by staff discussion of itemized problem lists, in order to systematically identify each underlying issue with the goal of preventing future error [1, 53]. However, it has been demonstrated in the aviation industry that this type of process is not adequate, nor ideal, to capture and respond to error, specifically related to system issues [14, 56]. Root cause analysis has been proposed as a means to identify system failures and look for potential solutions [2, 56]. Root cause analysis has been described in detail by Vincent et al. [57, 58]. Essentially, this type of analysis provides physicians with a more structured framework to improve patient safety. Their proposed framework is as follows: identification of the adverse event, why the event occurred (consisting of an analysis of different factors, related to the patient, the task, the caregiver team, the information technology, and the local and institutional environment), implementation of interventions to reduce the probability of its reoccurrence, and finally evaluation of the effectiveness of these interventions. Other methods of analysis have been proposed as well, such as the Association of Litigation and Risk Management (ALARM) method [58, 59]. The ALARM method is limited due to a lack of direct evidence in the literature; the usefulness of this analytical framework in MMC, although promising, has yet to be characterized.

## 5. Proposed Guidelines 

Based on the heterogeneous nature of the available literature, although it is difficult to synthesize evidence-based recommendations, some suggestions for the conduct of MMC can certainly be proposed.

### 5.1. Goals

The goals should be both quality improvement and education. The MMC should be organized such that an optimal balance is maintained at each MMC within a given department. It should also be noted that these goals are not always mutually exclusive and are often complimentary.

### 5.2. Structure

As there is no strong evidence for the frequency of MMC, the traditional frequency of monthly MMC in medical departments and weekly MMC in surgical specialties may be appropriate. Instead of commenting on a recommended frequency, we propose that each department evaluates whether monthly or weekly conferences are adequate for meeting the services' desired goals, while balancing the other priorities of the department (such as time management, resident education, and patient safety). Similar recommendations can be made with regard to the number of cases discussed or the duration of the MMCs. Arguably, the participants ought to be multidisciplinary, particularly as solutions to systematic problems, usually necessitate a multipronged approach. The presence of a formally recognized facilitator, who moderates the participation of the various members, would enhance MMC outcomes.

### 5.3. Process

Cases should be selected with predefined criteria or using other existing complication database registries. The cases selected should include both preventable and nonpreventable adverse outcomes, cases with opportunity for quality improvement, cases with educational values, or rare events. For each event presented, we suggest an analysis based on a framework such as the root cause analysis model, to improve effectiveness in identifying both individual and systemic factors. We also suggest appropriate incorporation of evidence-based medicine as well as initiation of quality improvement recommendations during these conferences.

## 6. Conclusion

Patient safety is of vital importance in the practice of medicine. Both medical and surgical services aim to improve patient safety through Morbidity and Mortality Conferences. Although there is a paucity of evidence with regard to their effect on hard outcomes, they are arguably a fundamental tool for achieving important goals in education and quality improvement. Using a unifying conceptual framework for the content and process of MMC, we attempt to summarize the existing literature in a simple, consistent, and reproducible fashion. It is clear that further research is needed to assess the use of different available frameworks to improve the effectiveness of MMC for both medical education and patient safety purposes.

## Figures and Tables

**(a):** 

Study/setting	Stated objective	Category	Type of study	Main results and conclusion
Kirschenbaum et al., 2010 [18] ICU Academic	Determining if audit of patients plus a focused MMC improved patient care in ICU	Goal	Interventional: before and after survey	MMCs result in improved rapid response and hospital outcomes (number of cardiac arrests decreased from 3.1/1000 to 0.6/1000, *p* = 0.002, deaths decreased from 34/1000 to 24/1000, *p* = 0.024).

Ksouri et al., 2010 [19] ICU Academic	Evaluating MMC in ICU for improving quality of care and patient safety	Goal, structure, process	Retrospective	MMCs provide educational value and can be used to assess quality of care, patient safety, and interpersonal and team communication.

Kuper et al., 2010 [20] Academic	Exploring the role of MMC in medical education	Goal, structure	Prospective/ethnographic: interviews, evaluation of notes, and audiotape of MMC	MMCs are effective vehicles to address competencies in patient safety and quality improvement. A disjunction between teaching valued by staffs and learning valued by students were noted.

Szostek et al., 2010 [21] Academic	Determining educational value of system audit	Goal, structure, process	Interventional: before and after survey	MMCs with system audit have higher educational values, 95% (versus 61% preimplementation) and stimulating increased interest in education as well as ensuring improved quality of care.

Bechtold et al., 2008 [22] Academic	Describing new MMC experience	Goal, structure, process	Interventional: before and after survey	New MMC format allows good educational forum with increased participation. Educational intervention and recommendations were more likely to be carried out.

Hasan and Brown, 2008 [23] Academic	Proposing a format as a model for MMC in academic center for gastroenterology	Structure, process	Prospective: chart review	Overall complication rate of 0.76%, within that reported in the literature. Monthly MMCs are a means of monitoring patient care and enhancing trainee education.

Goldszer et al., 2006 [24] Community	Describing MMC in primary care center	Goal, structure, process	Prospective	The MMC format is a useful tool to improve patient care.

Kravet et al., 2006 [25] Academic	Evaluating the role in teaching 6 competencies of ACGME with MMC implemented in Grand Round	Goal, structure	Cross-sectional: survey	MMCs in Grand Rounds are effective (well attended) and add diversity in topic and teaching methods.

Denis et al., 2003 [17] Community	MMC format assessed as a quality improvement tool in gastroenterology	Goal, process	Prospective: chart review	Systematic prospective recording of complications and careful exhaustive retrospective analysis during MMC are efficient and complementary tools for continuous quality improvement.

Esselman and Dillman-Long, 2002 [26] Academic	Refocusing MMC onto system issues and avoiding placing blame on individuals	Goal, structure, process	Retrospective	MMCs are important in quality improvement when focusing on system issues.

**(b):** 

Study	Setting discipline	Stated objective	Category	Type of study	Main results and conclusion
Falcone and Watson, 2012 [27]	Academic surgery	Assessing participation and cost benefit of teleconference in MMC	Goal, structure	Retrospective cost-effective analysis	Teleconferencing allows for increased faculty attendance at MMC (5 per conference, *p* < 0.001) and is cost-effective (annual net savings of 7624$).

Falcone et al., 2012 [28]	Academic surgery	Describing reporting patterns of general surgery residents. Describing adverse events rates compared to published data	Process	Retrospective cohort	Underreporting of nonfatal adverse events: 2.5% versus 4.3% reported in literature; majority of adverse events were from death (24.1%), hematologic or vascular complications (16.7%), and gastrointestinal complications (16.1%).

Thomas et al., 2012 [29]	Academic surgery	Integrating minor complication reporting in MMC for its educational value	Goal, structure, process	Interventional: before and after survey	Postimplementation of reporting of minor adverse outcomes in MMC; 95% of surveyed population (*p* < 0.01) stated that this provides improved quality assurance (71%, *p* < 0.05).

Bevis et al., 2011 [30]	Academic obstetrics	Characterizing the MMC as a cost-effective and efficient approach for addressing the ACGME competencies	Goal, structure	Retrospective	MMCs address 100% practice-based learning and medical knowledge, 19% systems-based practice, 10% communication, and 6% professionalism or ethics.

Kauffmann et al., 2011 [31]	Academic surgery	Multidisciplinary MMC presents a unique opportunity to incorporate all 6 ACGME competencies effectively and efficiently	Goal, structure, process	Retrospective	Multidisciplinary MMCs are useful in rapidly achieving quality improvement while creating opportunities for system health care delivery initiatives.

Kim et al., 2010 [32]	Academic surgery	Examining the content and process of MMCs and testing the hypothesis that a structured format can improve teaching and learning	Goal, structure	Interventional: before and after survey	A structured MMC format improves the identification of the cause for complication (3.11 to 4.56, *p* < 0.05). 67% of surveyed population expressed an overall improved experience in quality of care.

Steiger et al., 2010 [33]	Academic neurosurgery	Describing methods to identify critical cases, the system of analysis, classification of MMC, and resulted impact	Goal, process	Retrospective	A reliable system is employed by MMC to identify cases, providing good instruments for quality control and problem oriented teaching. Impact on quality improvement remains questionable.

Antonacci et al., 2009 [34]	Academic and community surgery	Describing comprehensive surgeon report card system based on MMC, in a nonpunitive error analysis fashion	Goal, structure, process	Prospective	MMCs result in a 40% reduction of gross mortality (*p* < 0.001). Quality issues were identified as 3 times greater than required by New York State regulations.

Berenholtz et al., 2009 [12]	Academic surgery	Describing learning from a defect tool as a strategy to meet ACGME requirements and enhance traditional MMCs	Goal, structure, process	Prospective	MMCs present a helpful strategy to learn from medical incident and improve patient safety and quality of care. Adverse events are usually failures in the system.

Bender et al., 2009 [35]	Academic surgery	Determining heterogeneity of assessment in peer-reviewed MMC and evaluating biases	Process	Prospective: survey	Significant disagreement noted amongst assessors leading authors to conclude that the reliability of peer review is questionable.

Dissanaike et al., 2009 [36]	Academic surgery	Comparing the perceptions of preventability of mortalities and severity of complications of MMC attendees	Structure, process	Prospective	Surgical residents assign higher severity to trauma-related complications than other groups. More objective grading tools are necessary to improve the adequacy of MMC.

Greco et al., 2009 [37]	Academic surgery	Describing the authors' experience with incorporating a clinical librarian into the process of MMCs	Goal, structure	Prospective	The clinical librarian program has improved the quality of MMC presentations.

Folcik et al., 2007 [38]	Academic surgery	Describing a two-tiered process MMC with dedicated subcommittee for quality improvement for ACGME competencies	Goal, structure, process	Prospective: reviewed MMC note, survey	MMCs with a dedicated quality improvement subcommittee decrease time to implementation of changes (3-4 months compared to 10–12 months).

Prince et al., 2007 [39]	Academic surgery	Analyzing which features of MMC associated with greater educational value and increasing confidence in the future	Goal, structure	Prospective: survey	Audience interaction improves educational value and increased confidence in managing complex problems presented in MMC (*p* < 0.01). This is achieved by increased questioning and explanation, radiology images read by presenters, and moderators facilitating discussion.

Goldfarb and Baker, 2006 [40]	Community surgery	Sharing a reproducible process for presenting, analyzing, and reducing surgical morbidity and mortality	Goal, structure, process	Retrospective: chart review	MMCs help in directing changes to resident training, hospital systems, and surgical practice.

Hutter et al., 2006 [41]	Academic surgery	Comparing data as reported in a traditional MMC versus National Surgical Quality Improvement Program (NSQIP)	Goal	Retrospective: MMC data reviewed	MMCs underreport adverse events when compared to NSQIP: 1/2 deaths and 3/4 complications were not presented, especially in patients with incurable disease, transferred care, and “medical” problems.

Miller et al., 2006 [42]	Academic urology	Comparing complications reported at the MMC versus NSQIP	Goal, process	Retrospective: chart review	MMCs have low sensitivity for detection of complications (25%). NSQIP may be better for urologic quality improvement endeavors.

Rosenfeld et al., 2005 [43]	Community surgery	Evaluating new MMC for ACGME competencies	Goal, structure, process	Retrospective: chart review	The restructuring of MMC so that a case is analyzed according to ACGME general competencies improved general interest and educational value. MMCs provide opportunities to teach ACGME general competencies.

Murayama et al., 2002 [44]	Academic surgery	Evaluating impact of changes made to our MMC (5–10 min case summary, literature review, and faculty discussion with moderator)	Goal, structure	Interventional: before and after survey	Surgical residents perceive significant improvements after changes to the MMC process. This is not the case for surgical staff.

Risucci et al., 2003 [45]	Academic surgery	Assessing interrater agreement before and after initiation of a modified MMC (presentation of 3 cases of 30 minutes with literature review)	Structure, process	Interventional: before and after survey	After modification of MMC, the majority of surveyed population perceives that consensus has been reached more often (96% of cases versus 70% cases *p* < 0.01) especially for avoidability of complications (54% of cases versus 23 of cases, *p* < 0.05).

Veldenz et al., 2001 [46]	Academic surgery	Determining educational value of MMC in surgical residency program	Goal, structure, process	Retrospective	A weekly peer-reviewed MMC provides educational value with ongoing examination of common problems encountered in the delivery of surgical care.

Hamby et al., 2000 [47]	Academic surgery	Determining the effectiveness of routine incorporation of local practice data in MMC	Goal, structure, process	Prospective: chart review	Incorporating prospective outcome data into the MMC provides increased educational values and opportunities for quality improvement.

Feldman et al., 1997 [48]	Academic surgery	Comparing the incidence of adverse outcomes recorded in a prospective general surgery database with that of MMC	Structure, process	Prospective: chart review	Although most severe complications (87.5%) are recorded at MMC, a large proportion of complications remain unreported. Rigorous monitoring of outcomes may contribute further to improvements in quality of care.

Thompson and Prior, 1992 [49]	Academic surgery	Determining the role and efficacy of surgical MMC in a current quality assurance program	Goal	Retrospective: chart review	Although many adverse events are not identified by MMC, these conferences remain an important component of quality assurance program.

Baele et al., 1991 [50]	Academic anesthesia	Describing the format of MMC in detail	Goal, structure, process	Prospective: chart review	MMCs offer a good educational role for residents through sharing of experiences, using a “no-blame” attitude. MMCs improve prevention of complications.

**(c):** 

Study	Setting	Stated objective	Category	Type of study	Main results and conclusion
Szekendi et al., 2010 [16]	Academic	Sharing the authors' experience with a patient safety oriented MMC over 7 years	Goal, structure, process	Interventional: before and after survey	Shift in staff perceptions of culture: increased voluntary reporting (by 66%), improved patient safety, and amelioration of quality of care.

Aboumatar et al., 2007 [2]	Academic	Describing MMC formats across multiple clinical departments; comparing MMC processes with previously published medical incident analysis models; and exploring how MMCs could be modified to advance medical education and improve patient care	Goal, structure, process	Cross-sectional: survey	MMCs vary in structure and process and fail to use known analytic framework. Well conducted MMCs provide valuable educational and quality assurance benefits. MMC should elicit input from all caregivers involved, follow a structured approach to identify system defects, and ensure adequate follow-ups on recommendations.

Pierluissi et al., 2003 [4]	Academic	Determining the frequency at which MMCs include adverse events and errors; determining whether errors are discussed and attributed to a particular case	Structure, process	Cross-sectional and prospective	Cultural difference between internal medicine and surgery noted. In internal medicine, fewer cases are presented (1.5 versus 2.7 cases, *p* = 0.001) but more time is spent on case presentation and discussion (34.1 minutes versus 11.7, *p* = 0.001). Fewer cases included adverse events (37% versus 72%, *p* < 0.001) or errors (18% versus 42%, *p* = 0.001).
